# Technical Skill Acquisition in Pediatric Minimally Invasive Surgery: Evaluation of a 3D-Printed Simulator for Thoracoscopic Esophageal Atresia Repair

**DOI:** 10.3390/healthcare13141720

**Published:** 2025-07-17

**Authors:** Sara Maria Cravano, Annalisa Di Carmine, Chiara De Maio, Marco Di Mitri, Cristian Bisanti, Edoardo Collautti, Michele Libri, Simone D’Antonio, Tommaso Gargano, Enrico Ciardini, Mario Lima

**Affiliations:** 1 Pediatric Surgery Department, IRCCS Sant’Orsola-Malpighi, Alma Mater Studiorum, University of Bologna, 40138 Bologna, Italy; sara-cravano@libero.it (S.M.C.); annalisa.dicarmine@gmail.com (A.D.C.); chiara.demaio@studio.unibo.it (C.D.M.); bisanticristian96@gmail.com (C.B.); michele.libri@aosp.bo.it (M.L.); simone.dantonio@aosp.bo.it (S.D.); tommaso.gargano2@unibo.it (T.G.); mario.lima@unibo.it (M.L.); 2 Pediatric Surgery Department, IRCCS Meyer, 50139 Florence, Italy; edocolla.ec@gmail.com (E.C.); enrico.ciardini@meyer.it (E.C.); 3 Faculty of Medicine, Alma Mater Studiorum—Università degli Studi di Bologna, 40126 Bologna, Italy

**Keywords:** minimally invasive surgery, pediatric surgery, thoracoscopy, esophageal atresia, simulation-based training, 3D printing, surgical education

## Abstract

**Background:** Minimally invasive surgery (MIS) is increasingly adopted in pediatric surgical practice, yet it demands specific technical skills that require structured training. Simulation-based education offers a safe and effective environment for skill acquisition, especially in complex procedures such as thoracoscopic repair of esophageal atresia with tracheoesophageal fistula (EA-TEF). Objective: This study aimed to evaluate the effectiveness of a 3D-printed simulator for training pediatric surgeons in thoracoscopic EA-TEF repair, assessing improvements in operative time and technical performance. **Methods:** A high-fidelity, 3D-printed simulator replicating neonatal thoracic anatomy was developed. Six pediatric surgeons at different training levels performed eight simulation sessions, including fistula ligation and esophageal anastomosis. Operative time and technical skill were assessed using the Stanford Microsurgery and Resident Training (SMaRT) Scale. **Results:** All participants showed significant improvements. The average operative time decreased from 115.6 ± 3.51 to 90 ± 6.55 min for junior trainees and from 100.5 ± 3.55 to 77.5 ± 4.94 min for senior trainees. The mean SMaRT score increased from 23.8 ± 3.18 to 38.3 ± 3.93. These results demonstrate a clear learning curve and enhanced technical performance after repeated sessions. **Conclusions:** Such 3D-printed simulation models represent an effective tool for pediatric MIS training. Even within a short time frame, repeated practice significantly improves surgical proficiency, supporting their integration into pediatric surgical curricula as an ethical, safe, and efficient educational strategy.

## 1. Introduction

Esophageal atresia (EA) is a rare congenital anomaly occurring in approximately 1 in 3500 live births, frequently associated with tracheoesophageal fistula (TEF). Surgical repair is the mainstay of treatment, yet it presents significant technical challenges due to the small operative field, fragility of neonatal tissues, and associated comorbidities [1]. Despite advances, postoperative complications such as anastomotic strictures, recurrent fistulas, and gastroesophageal reflux disease remain common and often require additional interventions [2].

In recent years, surgical education has undergone a significant paradigm shift from time-based models to competency-based frameworks. This evolution emphasizes the attainment of specific technical and cognitive skills before operating on real patients. Particularly in pediatric surgery, where many procedures are both complex and performed infrequently, simulation-based training offers a reproducible, risk-free, and ethically sound alternative to traditional methods such as live animal or cadaveric models. As recently highlighted, integrating simulation into structured surgical curricula helps bridge the gap between theoretical learning and operative performance, promoting both safety and proficiency among trainees [3].

Minimally invasive surgery (MIS) has increasingly become the standard of care for a wide range of surgical conditions [4]. Numerous studies have shown that both laparoscopic and robotic approaches reduce postoperative pain, minimize the need for analgesics, accelerate recovery, shorten hospital stays, improve cosmetic outcomes, and lower the risk of adhesion formation [5,6,7,8]. These advantages are even more significant in pediatric patients, where minimizing surgical trauma is especially critical [9]. The growing application of MIS in pediatric surgery necessitates the use of specialized instruments and technologies, along with appropriate training for healthcare professionals [10,11,12].

The transition from open surgery to MIS demands advanced technical abilities, such as depth perception, hand–eye coordination, and precise instrument manipulation. Simulation-based training, encompassing both basic and complex procedures, plays a vital role in educating surgical trainees and refining the skills of experienced surgeons [13,14]. Simulation provides a safe, controlled environment where learners can repeatedly practice without posing any risk to patients [15]. Evidence supports that competencies acquired through simulation translate into improved performance in the operating room [16]. Repetitive and structured training sessions significantly contribute to the development and enhancement of motor skills [17,18].

To address the limitations of traditional training—such as cadaveric dissection and live animal models, which raise ethical, financial, and logistical issues [19]—simulation-based education offers a viable, risk-free alternative. The advent of 3D printing technology has enabled the creation of high-fidelity anatomical models, providing an effective and ethically sustainable solution for surgical training [20].

This study aims to assess the efficacy of a 3D-printed simulator designed for training in thoracoscopic repair of esophageal atresia with tracheoesophageal fistula (EA-TEF). Specifically, it evaluates the impact of repeated, short-term simulation on operative efficiency and skill acquisition among pediatric surgical trainees. Ultimately, this study underscores the critical importance of structured simulation training as an integral part of surgical education, ensuring that future surgeons acquire the necessary technical competencies and confidence to manage complex procedures effectively. In conclusion, the objective of the study was to assess the performance improvement of pediatric surgeons using a synthetic simulator specifically designed for thoracoscopic repair of esophageal atresia with tracheoesophageal fistula (EA-TEF).

## 2. Methods

This study was conducted at the Simulation Center for Minimally Invasive Pediatric Surgery of IRCCS Sant’Orsola-Malpighi, Bologna, from 27 March to 22 April 2024.

### 2.1. Simulator

**Figure 1:**
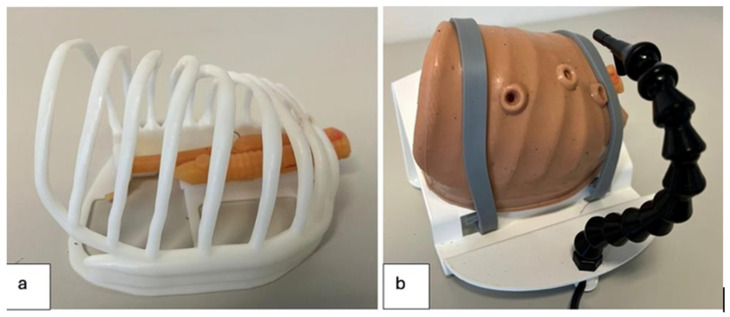
Three-dimensional trainer model used for simulation of esophageal atresia repair; (**a**) Thoracic cage with 3D atretic esophagus with distal TEF model inside; (**b**) Silicon 3D model of neonate’s chest with access point for thoracoscopic procedures.

The simulator consisted of a synthetic trainer model developed through 3D printing technology, replicating the neonatal thoracic cavity and EA-TEF anatomy (Figure 1).

**Figure 2:**
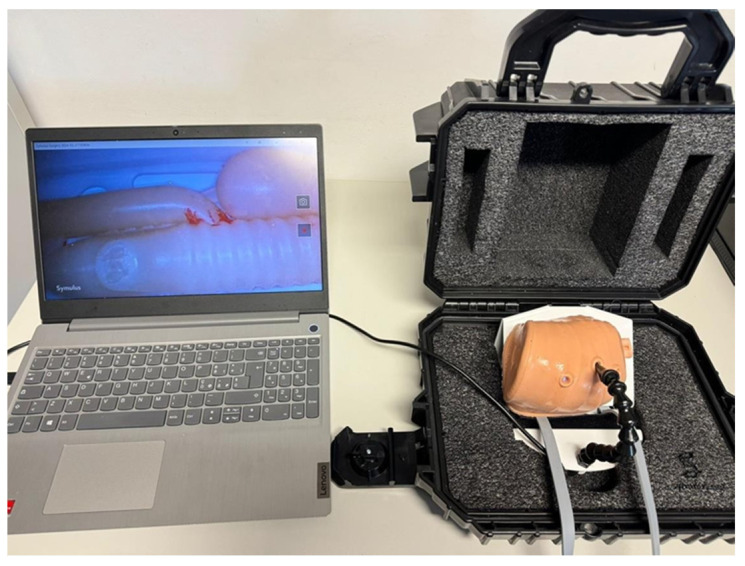
Trainer box with 3D neonatal chest connected to computer screen for the visualization of thoracoscopic images.

Its main components included:

- Thoracic cage: Modeled from a CT scan of a neonatal chest and scaled to 1:1.10.
- Silicone skin: A 3D-printed mold was used to produce a silicone layer that fits over the thoracic cage, with three access ports for thoracoscopic instruments and optics. The lateral ports allowed insertion of laparoscopic instruments (needle holders and scissors), while the central port was dedicated to the optical system.
- Optical sensor and lighting system: A PMW3360 optical sensor, connected via USB to a computer, provided real-time video feedback from within the thoracic cavity (Figure 2) [19].

**Figure 3:**
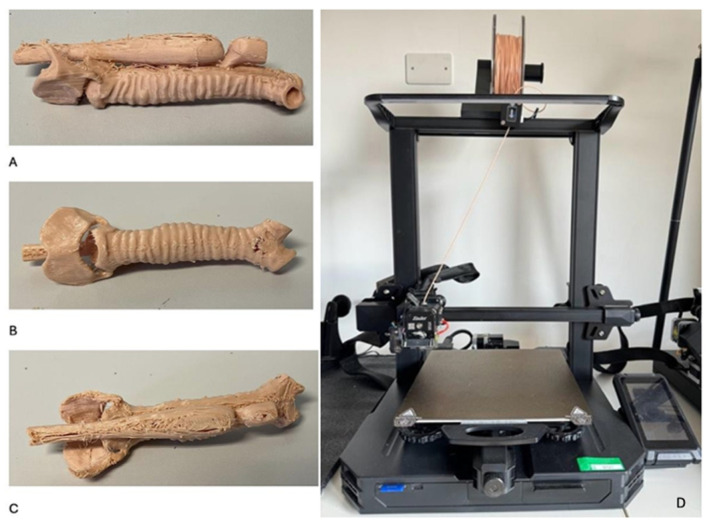
(**A**) The 3D model of atretic esophagus with the two esophageal stumps and fistula between the distal one and the trachea; (**B**) The 3D-printed model of trachea with thyroid and cricoid cartilages; (**C**) Posterior view of 3D-printed model of trachea and the two esophageal stumps; (**D**) The 3D printer used for the creation of the model applied in the study.

The three-dimensional model of the atretic esophagus with distal TEF was printed using the Ender 3 (Creality), a fused deposition modeling (FDM) printer. This process created a highly detailed, life-sized anatomical model, including both esophageal stumps, the distal fistula, and relevant tracheal anatomy (Figure 3).

### 2.2. Surgical Instruments

**Figure 4:**
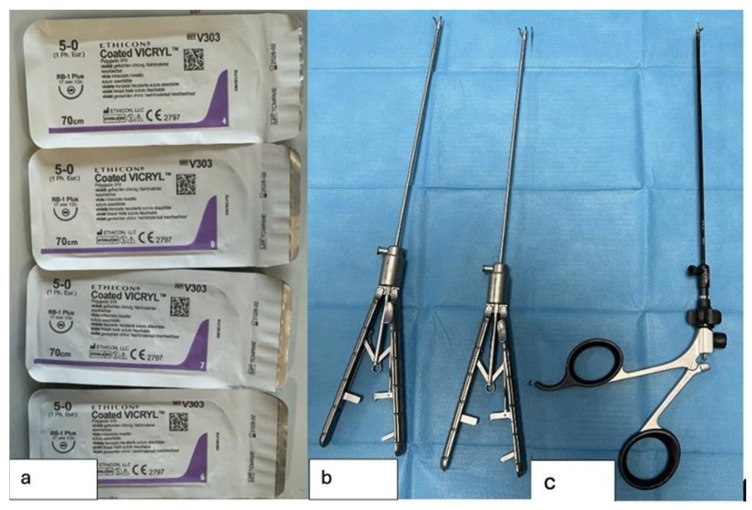
Surgical instruments used in the study; (**a**) Sutures used for TEF ligation and esophageal anastomosis; (**b**) Laparoscopic needle holders; (**c**) Laparoscopic scissors used to dissect the TEF and opening the esophageal stumps.

The procedures were performed using the following instruments (Figure 4):

- 4 Vicryl 5-0 sutures with a 17 mm needle
- 2 laparoscopic needle holders
- 1 laparoscopic scissors

### 2.3. Study Participants

Six pediatric surgeons from the Sant’Orsola Pediatric Surgery Unit were enrolled:

- 1 specialist pediatric surgeon with <5 years of experience
- 2 senior trainees (fourth-year residents in pediatric surgery)
- 3 junior trainees (third-year residents in pediatric surgery)

Participation was voluntary. All participants completed eight simulation sessions using the 3D-printed trainer.

### 2.4. Training Protocol

Prior to the simulation sessions, participants watched a standardized instructional video demonstrating the thoracoscopic EA-TEF repair. The video featured an expert pediatric surgeon performing the procedure under identical simulation conditions. This served to introduce the procedural steps, set performance expectations, and provide a benchmark for comparison.

Each participant performed the following four key steps:

- Closure of the tracheoesophageal fistula with a through-and-through suture
- Division of the fistula
- Opening of the proximal esophageal stump
- Esophageal anastomosis using interrupted sutures

Each session involved a total of 9 sutures: 1 for the fistula ligation and 8 for the anastomosis (2 at the 180° mark, 3 posterior, 3 anterior). Each suture was secured with 4 knots to ensure tension and stability.

### 2.5. Assessment and Data Collection

Two main metrics were recorded for each session:

- Operative time: From the beginning of the procedure to the final suture.
- SMaRT score (Stanford Microsurgery and Resident Training Scale): A validated tool assessing nine categories (instrument handling, tissue respect, efficiency, suture management, suturing technique, knot quality, final product, workflow, and overall performance), each scored from 1 to 5, for a maximum of 45 points (Table 1) [21].

An experienced minimally invasive pediatric surgeon observed all simulations and assigned the SMaRT scores to ensure objectivity. Data on both timing and scoring were entered into a purpose-built Excel database.

### 2.6. Study Duration and Objective

The training took place over 27 days. Each participant completed 8 simulations. The study aimed to track performance improvement in terms of time efficiency and technical precision across repeated sessions, providing a structured evaluation of surgical skill acquisition.

### 2.7. Statistics

Descriptive statistics were used to summarize operative times and SMaRT scores across simulation sessions. Continuous variables were expressed as mean ± standard deviation. To assess the improvement in operative times across simulations, data from the first and eighth sessions were compared. The Shapiro–Wilk test indicated a non-normal distribution of differences (*p* = 0.036). Therefore, in addition to the paired Student’s *t*-test (*t* = 13.70, *p* < 0.0001), the Wilcoxon signed-rank test was performed, confirming a statistically significant reduction in operative time (*p* = 0.031). Statistical analyses were performed using Microsoft Excel (Microsoft 365, Redmond, WA, USA) for descriptive statistics and paired *t*-test. The Shapiro–Wilk and Wilcoxon signed-rank tests were performed using Python (v3.10, SciPy library).

## 3. Results

The first simulation was performed by a specialist in minimally invasive pediatric surgery. The time required to complete the procedure—from the initial ligation of the tracheoesophageal fistula to the final suture of the esophageal anastomosis—was recorded as 61 min. All operative times were documented in minutes using a dedicated Excel database (Table 2).

### 3.1. Time Efficiency Improvement

**Figure 5:**
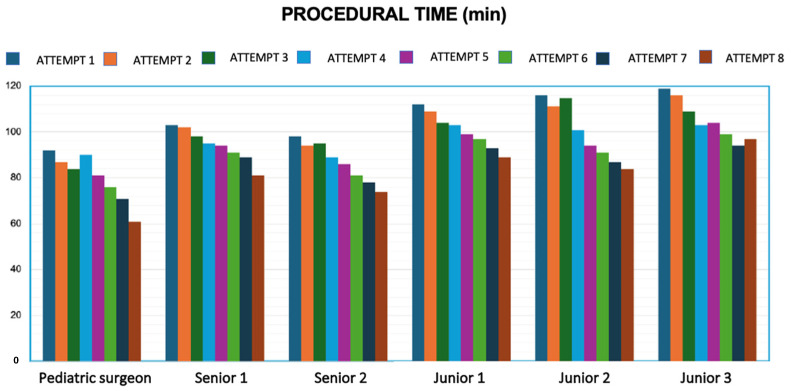
Progression in operative time for each participant from the 1st to the 8th simulation session.

A clear trend toward reduced operative time was observed over the course of the eight simulations for all participants. To quantify this improvement, the difference between the duration of the first and the eighth simulation was calculated for each surgeon (Figure 5).

In the first simulation, the average times recorded were:

- Specialist surgeon: 92 min
- Senior trainees: 100.5 ± 3.55 min
- Junior trainees: 115.6 ± 3.51 min

**Figure 6:**
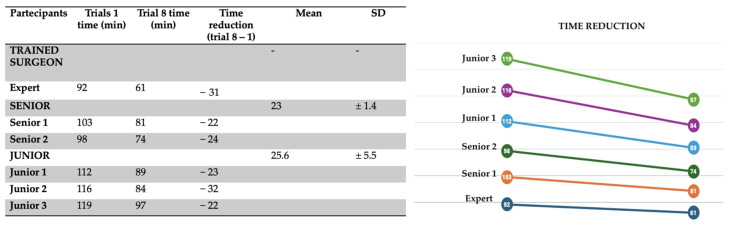
Variation in execution times across the eight simulations, demonstrating a trend of progressive improvement.

By the eighth simulation, these times had significantly improved (Figure 6):

- Specialist surgeon: 61 min
- Senior trainees: 77.5 ± 4.94 min
- Junior trainees: 90 ± 6.55 min

### 3.2. Technical Skill Assessment

**Figure 7:**
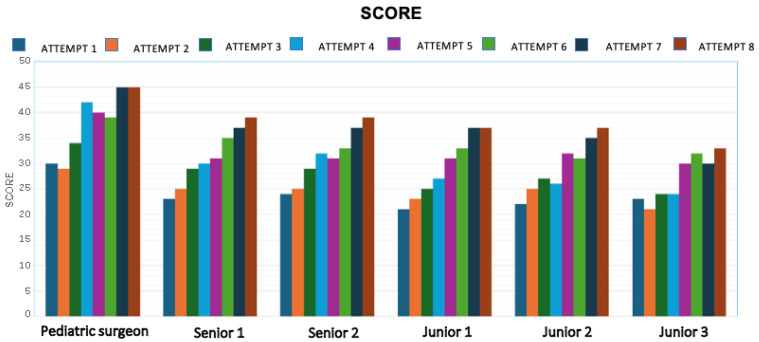
Trend in SMaRT scores for each participant across simulations, indicating improvement in technical performance.

Technical performance was evaluated using the SMaRT Scale, which includes nine parameters scored on a 5-point Likert scale (1 = failure, 5 = excellent). Each simulation was scored by the same experienced pediatric surgeon to ensure consistency and objectivity. As shown in Figure 7, a steady increase in the total SMaRT score was observed over time for all participants.

**Figure 8:**
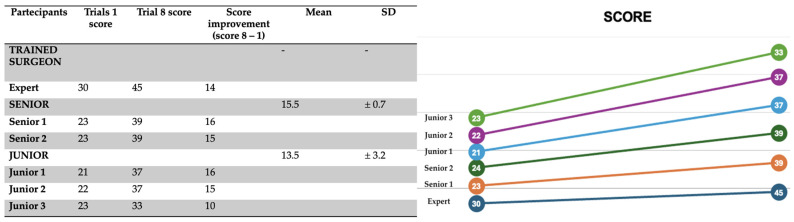
Change in average SMaRT score from the 1st to the 8th simulation, illustrating a clear upward trend in technical proficiency.

When comparing the average scores across all participants between the first and last simulations, the improvement is substantial (Figure 8):

- Simulation 1: 23.8 ± 3.18
- Simulation 8: 38.3 ± 3.93

## 4. Discussion

This study aimed to evaluate the effectiveness of a 3D-printed simulator specifically developed for training in thoracoscopic repair of esophageal atresia with tracheoesophageal fistula (EA-TEF). In particular, it focused on how repeated exposure to simulation over a short period could enhance operative efficiency and technical proficiency, especially among less experienced pediatric surgeons.

The findings confirm that repeated practice of a specific minimally invasive procedure leads to significant improvements in both operative time and procedural quality. These results are consistent with previous literature. For instance, Borselle et al. demonstrated that a short but intensive simulation program could significantly improve suturing skills for EA-TEF repair [22]. Similarly, Hong et al. created a 3D-printed model using both FDM and PolyJet technologies and validated its realism and training utility by comparing various materials [23]. Servi et al. reported the effectiveness of a 3D model for thoracoscopic pulmonary lobectomy training, highlighting anatomical accuracy and procedural fidelity as key benefits [24]. Deie et al. emphasized the added value of condition-specific simulators over traditional dry boxes, especially for rare neonatal conditions [25]. Youn et al. further confirmed the usefulness of patient-specific reconstructions, which enhanced tactile feedback and improved anastomotic technique [26]. Zahradnikovà et al. demonstrated the value of a 3D-printed model for thoracoscopic repair of esophageal atresia by analyzing the results achieved by medical students, pediatric surgical trainees, and expert surgeons [27].

Multiple applications of 3D-printed models in the simulation of minimally invasive pediatric surgical procedures have been reported in the literature. For instance, Williams et al. [28] reported results from a simulated training model for laparoscopic pyloromyotomy. Similarly, Cabarcas Maciá et al. [29] developed a 3D model for pediatric laparoscopic pyeloplasty simulation, and Heo et al. [30] designed a 3D-printed model for pediatric inguinal hernia repair training.

Similarly, recent developments by Petra et al. emphasized the potential of patient-specific anatomical models for enhancing procedural realism and preoperative planning [31]. However, our model uniquely integrates real-time optical feedback and a validated scoring system (SMaRT), allowing for structured assessment of technical progression.

Compared to traditional dry boxes, the 3D-printed model offers superior anatomical fidelity, particularly in replicating neonatal thoracic structures and the spatial constraints of thoracoscopic procedures [32]. While virtual simulators provide advanced visual guidance, they often lack the haptic feedback necessary for fine motor skill acquisition [33]. Unlike animal or cadaveric models, which pose ethical and logistical challenges, 3D printing enables reproducible, patient-specific simulations that are both cost-effective and customizable [34].

In our study, the progression of both operative time and SMaRT scores clearly demonstrates the steep learning curve of thoracoscopic EA-TEF repair when supported by simulation. Initially, more experienced surgeons outperformed trainees in terms of time and quality. However, after only eight sessions, all participants—regardless of prior experience—showed substantial improvements. This suggests that simulation training can accelerate the acquisition of advanced surgical skills.

Several factors contributed to this learning curve:

- Increased familiarity with thoracoscopic instruments and port placement
- Improved hand–eye coordination and two-dimensional spatial interpretation
- Adaptation to the absence of tactile feedback, typical of MIS procedures
- Standardization of technique through visual and procedural benchmarking

The gradual increase in SMaRT scores—particularly in categories such as instrument handling, suturing technique, and workflow—indicates a qualitative enhancement of surgical execution. The results showed consistent improvements in both surgical technique and operative timing across all participants. Between the sixth and eighth sessions, further gains were noted, although not statistically significant. This plateau effect may be attributed to several factors, including ergonomic limitations, task repetition, or cognitive saturation. Importantly, all these improvements were achieved without any risk to patients and in a controlled, reproducible environment. Notably, even the experienced pediatric surgeon exhibited measurable performance gains over the course of the training. This suggests that simulation-based training may benefit not only trainees, but also experienced surgeons who may initially be challenged by the unfamiliar ergonomic constraints of the simulated setting.

These findings support the integration of simulation-based training into pediatric surgical education programs. The use of 3D-printed anatomical models offers several advantages: reproducibility, anatomical accuracy, and cost-effectiveness. Unlike cadaveric or animal models, 3D simulators can be tailored to represent patient-specific anatomy, making them valuable not only for training but also for preoperative planning.

### Limitations of the Study

This study has several limitations. First, the small sample size limits the generalizability of the results and may not fully represent the variability in learning curves across different institutions. Second, performance was assessed in a simulated environment, which may not fully replicate the complexity and stress of real surgical settings. Third, the absence of inter-rater reliability assessment in the scoring process. Although a single experienced evaluator was used to ensure consistency, future studies should incorporate multiple independent raters to improve objectivity and allow statistical measurement of inter-rater agreement. Finally, the study evaluated only short-term improvements.

## 5. Conclusions

This study demonstrates that simulation-based training using 3D-printed models significantly improves technical skills and operative efficiency in pediatric minimally invasive surgery. Even after a limited number of sessions, participants showed marked progress in both precision and time management. These findings support the integration of high-fidelity simulation into surgical training programs as a safe, effective, and ethically sound method to enhance surgical competence and confidence.

## Figures and Tables

**Table 1:** SMaRT Scale (Stanford Microsurgery and Resident Training Scale) [21].

Category	Score 1	Score 2	Score 3	Score 4	Score 5
Instrument Handling	Unable to control instruments	Frequent errors in handling	Basic control with frequent corrections	Good control with minor errors	Excellent and smooth handling
Tissue Respect	Excessive tissue damage	Moderate damage	Occasional trauma	Minimal trauma	No trauma
Efficiency	Disorganized, inefficient	Frequently slow and unsure	Average performance with delays	Mostly smooth	Highly efficient and smooth
Suture Management	Unable to manage suture	Frequent tangling or misplacement	Some control but inconsistent	Good suture control	Excellent and consistent control
Suturing Technique	Incorrect technique	Frequent technical errors	Correct technique with inconsistencies	Mostly correct technique	Perfect suturing technique
Knot Quality	Knots frequently fail	Loose or insecure knots	Acceptable but inconsistent knots	Secure knots with minor variation	Strong, secure, and consistent knots
Final Product	Unacceptable outcome	Marginal quality	Adequate but needs improvement	Good quality	Excellent surgical result
Workflow	No clear plan, frequent interruptions	Inconsistent flow	Some planning, occasional hesitations	Organized and smooth flow	Fluent and anticipatory workflow
Overall Performance	Unable to complete task	Requires significant assistance	Completes with some guidance	Completes independently with minor issues	Outstanding independent performance

**Table 2:** Operative time per attempt for each participant (min = minutes).

Population	Attempt 1	Attempt 2	Attempt 3	Attempt 4	Attempt 5	Attempt 6	Attempt 7	Attempt 8
Pediatric Surgeon	92	87	84	90	81	76	71	61
Senior resident 1	103	102	98	95	94	91	89	81
Senior resident 2	98	94	95	89	86	81	78	74
Junior resident 1	112	109	104	103	99	97	93	89
Junior resident 2	116	111	115	101	94	91	87	84
Junior resident 3	119	116	109	103	104	99	94	97

## Data Availability

The original contributions presented in this study are included in the article. Further inquiries can be directed to the corresponding author.

## Abbreviations

MIS	Minimally Invasive Surgery
EA	esophageal atresia
TEF	Tracheoesophageal fistula
FDM	Fused Deposition Modeling
